# Between individualism and social solidarity in vaccination policy: the case of the 2013 OPV campaign in Israel

**DOI:** 10.1186/s13584-016-0119-y

**Published:** 2016-12-21

**Authors:** Hagai Boas, Anat Rosenthal, Nadav Davidovitch

**Affiliations:** 1 The Edmond J. Safra Center for Ethics, Tel Aviv University, Tel Aviv, Israel; 2 The Department of Health Systems Management, Faculty of Health Sciences, Ben-Gurion University of the Negev, Beersheba, Israel

## Abstract

**Background:**

During the summer of 2013, after samples of poliomyelitis virus were found in sewage, Israel launched an intensive national oral polio vaccine (OPV) campaign. The clinical objective of the campaign was rather clear. With not a single case of infantile paralysis and with a population already highly protected with IPV (a dead version of the vaccine), the goal was to foster collective immunity so that risk populations could also be protected. This, however, entailed a rather unusual issue: how to persuade parents whose children already received an IPV to re-vaccinate their children, now with a live yet attenuated version of the virus that was excluded from the national vaccination program in 2004. The challenge therefore was a call for social solidarity - asking parents to vaccinate their children mainly for the sake of protecting unknown at risk populations and to take part in the larger global goals of the polio eradication program. This challenge stands at the core of our investigation. We see the OPV campaign of summer 2013 as a good case study of the tension between individualism and social solidarity in seeking the cooperation of the public.

**Methods:**

We draw on a qualitative study that included participant observation, document reviews and interviews with policy-makers, parents, journalists, public health experts and community leaders. These data were analyzed in order to unravel the ways in which self-interest, community and solidarity were conceived by different agents during the vaccination campaign.

**Results:**

The family as a metaphor for social solidarity was the main discursive item in the public campaign. Tensions, dissonances and inconsistencies were found between different registers and agencies as to what is at stake and what is required.

**Conclusions:**

We discuss the ethical and social implications of our findings in order to better understand how persuasion was used in the current case and for its future role in similar events, within and outside Israel, when global efforts to eradicate polio are ongoing.

## Background

In response to the wild polio virus importation to Israel in 2013, the Ministry of Health decided to take preventive action by giving the oral polio vaccine (OPV) to all children born after January 1^st^, 2004, who had received at least one dose of inactivated polio vaccine (IPV) in the past. Apart from serving as a booster to increase individuals’ immunity, the main public health purpose of the campaign was to stop the potential environmental circulation of the wild virus. OPV—a vaccine that includes live, attenuated (weakened) poliovirus strains—is currently being used in mass vaccination campaign mainly in endemic countries. The vaccination offers both individual protection, mainly at the gastro-intestinal level (thus diminishing feco-oral transmission), and also protects against environmental transmission that boosts population immunity. This campaign was initiated by the Israeli Ministry of Health as a response to the findings of the virus samples in sewage in May 2013. However, it was not detached from the broader context of the global efforts to eradicate polio worldwide.

Both the WHO and the Israeli Ministry of health agreed that the Israeli 2013 polio campaign was unique, and it soon became clear that it bears importance to the “end game” period of polio eradication. It should be noted that the Israeli campaign was launched as response to the circulation of wild polio viruses as no clinical cases were found. The absence of clinical cases could be explained by pre-existing high vaccination coverage of the Israeli population. Nevertheless, since polio eradication efforts are global, and thus are not limited to states and involve the Global Polio Eradication Initiative and the WHO, Israel was advised to initiate a mass OPV campaign, similar to campaigns in polio endemic countries, thus reintroducing OPV to Israel.

The main question this article addresses concerns the challenge of persuading parents to vaccinate their already immune children, while reintroducing a vaccine that had been taken out of the national immunization schedule in 2004. The target population for vaccination was children up to 9 years old. Public health policy-makers met this challenge by introducing the family as a reference unit for health concerns, rather than the children who were targeted as the vaccinations’ recipients. “Two drops and the family is protected” became the campaign slogan used on television, ads and social media. In what follows we wish to focus on the implications of this campaign, and to understand its underpinning both analytically and pragmatically. We inquire about the image of society that was promoted by the campaign, its advantages and limitations, and what can be learned from this campaign in terms of future vaccination campaigns in Israel and elsewhere.

### Vaccinations, policy and social solidarity

Public health policies often stand at odds with our contemporary zeitgeist of individualism. Whereas individualistic conceptions place personal self-gain as both incentive for action and a desired result, public health policies address the personal self-gain as the end result of a collective benefit. Vaccinations are perhaps the paradigmatic example of this interplay. Individuals calculate whether or not to be vaccinated by considering their own self-interest in relation to the type and quantity of vaccines to which they are ready to be exposed. Public health policy-makers, in contrast, order vaccination programs by applying a set of considerations that extends the individual level and refers to the group, to the collective, as their main reference unit [2, 3, 5, 7]. In contrast to the personal balance of risks and benefits that individuals weigh when considering vaccinations, policy-makers think of vaccinations in terms of “herd immunity”, vaccination rates, and consider individual self-gain as a predictive outcome of the public good.

In the various public health ethical codes, solidarity is one of the foundations of public health practice, in the context of understanding humans as interdependent within communities—both at the national and global levels.^1^ Solidarity is especially used in cases of emergencies, persuading communities to take collective action and to suspend self-gain in favor of promoting collective good. This could be the case in collective responses in cases such as pandemics, for instance.^2^

Public health ethics scholars Angus Dawson and Marcel Verweij argue that although solidarity is being invoked as an important component in the success of public health policies in general, and vaccinations policies in particular, it is “remarkable that the concept of solidarity has received so little discussion in the bioethics literature.” [11] Thus a better understanding of how solidarity is being used not just as a normative term, but in practice, within a specific context, as in our case study, can help health policy makers and public health practitioners respond to emergencies in a more nuanced way. Persuading individuals and communities to vaccinate, using different incentives has been researched extensively in health literature. Yet, a quick search in PubMed for “vaccination” and “solidarity” yields only 22 publications, where a search for “vaccination” and “incentives” yields 635 publications.^3^

Current literature suggests that persuasion of populations, an important tool for public health practice that should be balanced with coercion [2], should take into consideration not just incentives given on individual levels, but the consideration of the notion of social solidarity as well [3, 5, 21]. Policy makers should note that a structural contradiction between the collective referential unit of public health policies and its application to individualistic modes of agency in the case of financial or other individual oriented incentives in order to obtain compliance, leads to dissonances that may impede the implementation of public health policies. In this respect, the following analysis provides a line of thought for policy makers that explores social structure as an important component in vaccination policy.

According to Prainsack and Buyx [21] social solidarity is defined as “manifestations of the willingness to carry costs to assist others with whom a person recognizes sameness or similarity in at least one relevant respect.” Solidarity, a value mentioned in various public health ethical codes of major public associations such as the American Public Health Association, European Public Health Association as well as the Israeli Association of Public Health Physicians, is not merely an abstract concept—it has public health policy implications and it points to the need to be more aware of the interplay between individualism and social structures. In the US, scholars have been discussing the unfashionable place of solidarity in the American value system. In the context of the Affordable Care Act (Obamacare) debates, attempts were made to introduce solidarity in a way that reflects “American nature”, and interpreted to include within solidarity issues such as mutual assistance, patriotism and coordinated investment. Thus some American scholars and policy analysts are trying to promote solidarity as a legitimate term, while strengthening more individualistic and market oriented values [23, 24].

During the OPV campaign in Israel in the summer of 2013, the need for collective action which does not directly benefit the vaccine’s recipient himself became apparent. In what follows we address the different ways in which concepts of “society”, “solidarity” and “individualism” were used and modified by different parties, how they were used to persuade and evoke compliance, and how policy makers and the public understood them in practice during the unfolding events.

### The challenge

The public health objective of the OPV campaign in Israel was rather clear. With not a single case of infantile paralysis, and with a population already highly protected with IPV, the goal was to boost immunity on the individual level, but more importantly to foster collective immunity so that the wild virus will stop circulating and that populations at risk would also be protected.^4^ This, however, entailed an important goal: to convince parents whose children already received an IPV to re-vaccinate their children, now with a live attenuated version of the virus; the same vaccine that was taken out of the national vaccination program in 2004, due to its higher, though rare, risk of side effects. Administration of OPV is associated with a low incidence of paralytic poliomyelitis in vaccines (approximately 1 case per 2.6-7 million doses of OPV administered, which is reduced to a minimum if given after being vaccinated with IPV; no such incidents were recorded in this campaign). Also, individuals in close contact with recently inoculated vaccines may be at a small risk of developing paralytic poliomyelitis because poliovirus can be shed in the feces (and possibly from the pharynx) for 6–8 weeks after OPV administration. Immunocompromised patients are also susceptible to this adverse reaction. The challenge therefore was to call for a non-egoistic behavior for the benefit of society: taking the time and effort to go to a clinic for vaccination and exposing one’s child to a vaccine (even if the risk is minimal to the point of being practically non-existent) and possibly immunocompromised family members for the sake of protecting an unknown group of people.

This challenge was further complicated by the fact that the polio vaccination campaign in Israel could not be separated from the efforts made by the larger global eradication campaign, thus receiving international scrutiny from various stakeholders, mainly from the WHO and the Global Polio Eradication Initiative. The reintroduction of polio also meant that Israel, as part of the European WHO region, might change the European status of a polio free region. From that perspective, solidarity has a much more global meaning, as it refers to the role of various countries in achieving the global goal of eradication, a perspective that is often unaddressed in the local national discourse.

These challenges stand at the core of our investigation. We see the OPV campaign of summer 2013 as a good case study to better understand if and how solidarity was evoked to persuade parents to vaccinate their children with OPV. Since compliance to public health interventions is based on a variety of values, among them solidarity, social responsibility and trust, exploring the 2013 polio campaign in Israel—as presented by policy makers, public health professionals and the public—can provide us with a better understanding of vaccination policy making, and responses to public health emergencies.

## Methods

This article draws on evidence collected during a qualitative study of the public reaction to the 2013 Israeli polio campaign. Data collection was conducted during the summer and fall of 2013, while campaign activities were ongoing, and the winter of 2014, after the campaign’s conclusion. Since qualitative methods have been shown to be a beneficially valid tool of inquiry in public health, especially when complex beliefs and experiences are concerned [26], this study included 16 open-ended interviews with ministry of health officials, journalists, and health practitioners including nurses, pediatricians and parents. We have obtained consent from all participants to use the views and perspectives they expressed in the interviews, while revealing their professional identity and concealing their personal identity. We have coded the interviews according to dominant themes that were of analytic interest to us. In addition, we have conducted participant observation in campaign activities, and collected newspaper articles, opinion columns, blogs, and official publications of the ministry of health as they appeared on the ministry’s website, and other electronic media, during the summer of 2013. We have coded these qualitative data in a discourse analysis method, where we indicated the main themes and emphases that were introduced in the different media during the OPV vaccination campaign.

## Results

### The individual, the community, and the Israeli state

Not only did the polio campaign result from Israel’s unique policy of standardized monitoring of sewage for traces of polio viruses [20], we also argue that both the campaign and the public reaction to it are rooted in Israeli idiosyncrasy. Indeed, public reaction to vaccination cannot be understood without addressing the complex political landscape of Israeli society. And while the local landscape is an important factor when addressing vaccine refusals in every society [4, 7], the particularities of Israeli society shaped the local patterns of acceptance and refusal on the one hand, and of the campaign methods on the other.

Fostering trust in the system, as in any public health campaigns, became the prime goal of the polio vaccination campaign. Trust is often achieved as a result of a transparent and consistent policy. In this case, the reintroduction of a vaccine that was excluded from routine immunization protocols only a decade ago, required clear answers. However, this move—although clear to policy-makers and public health experts—resulted in suspicion among non-health professionals.

The campaign leaders sought a way of persuading the public to act for the benefit of others and needed therefore to conceptualize these others in a language that would induce compliance. However, the meaning and use of terms such as the “Public” or “Society”, have been constantly changing in the context of rapid process of privatization in healthcare in Israel. In order to cope with growing healthcare expenditures and heightened budget constraints, many countries (including Israel) have adopted various models of public-private partnerships. Privatization of healthcare can be reflected in various ways: modes of financing, service delivery and changing modes of operation adapted more to for-profit schemes [10]. Privatization of healthcare in Israel did not occur in a void. Starting from a more collectivistic perspective that was prevalent in the 1950s, widespread American influences have eroded “the public” or “society” to the point where political schemes are structured as the dichotomy of the individual vs. the state ([22]; on the relationship between individualism and privatization in healthcare see also: [9]). Noting these changes in Israeli society, a public health physician offered his observations on the impact they had in recent decades:

*“Something very basic has changed in Israel…. The health system turned from paternalistic to less paternalistic, but specifically in Israel there was a major change in my mind, in people’s commitment to the community, to the state. Solidarity that might have been more focused on the state than on the community in the past, has gone down and now it’s more the issue of “me”, individualism, how does it benefit me. And once that’s the case, everything the state wants from you, you check if it’s worthwhile for you personally. You immediately check everything, everything makes you suspicious”.*

Social forces emphasizing individualism as well as a massive push toward the privatization of health services had shaped the public reaction to the campaign. Individual citizens experienced these processes of privatization not just as the state’s disengagement from their health, and the health of their children, but also ultimately as a professional failure on part of the health system. This failure to provide services had eroded the trust between the state and its citizens. The same public health physician explained the context in which these failures occurred:

*“I think the real story is how health services for school children were privatized. In recent years the state had privatized these services to bodies who, even though they received more money than the public system would have spent, were unable to perform the tasks, and did much less in terms of health promotion [in school children]…. And now when parents get a letter [about children’s health] from someone, who is a for-profit company being paid by the ministry of health, parents can be suspicious.”*

Commenting on the effects of privatization of health services on citizens’ trust in the state, a senior ministry of health public health physician commented:

*“The public is skeptic when it comes to the interests of the establishment. With empty slogans, they [the representatives of the state] are putting their hands deeper and deeper into our pockets. Yes, we are the ministry of health, and we see ourselves as the good guys, but the public sees us as part of the establishment. They don’t see us as the good guys. We are seen as just another government office, giving a government order. And people object to that, they are skeptical about that.”*

The above quotes reflect the social implications of these shifts to individualism and privatization: even when it comes to one’s health, that was once the responsibility of the state, the individual feels that he or she are no longer receiving the same kind of support from the state. And from that perspective, personal survival becomes the name of the game.

In this state of affairs, self-interest becomes the main orientation of practice. Far from group solidarity, self-interest begets mainly suspicion and mistrust. And while many expressed suspicions regarding the nature and necessity of the vaccination campaign, concerns were negotiated through various mechanisms. Addressing this suspicion, a senior Ministry of Health public health physician explained:

*“There were questions (about the OPV vaccine), and it’s a bit weird that there were questions, because until 2005 it was given as routine [immunization] and there were no questions… The (changes in) policy confused people. But more than that, the public is not getting smarter, it’s getting pickier and skeptic… first the establishment is not seen as reliable… and the public is skeptic about the establishment’s agenda”*.

Following an explanation of how trust in government ministries and officials, has dwindled due to the sense that the government’s agenda is less reliable, the same senior ministry of health public health physician has said (referring to the vaccination coverage rates known at the time of the interview):

*“And yet, at the end a voluntary coverage of over 60% of the children is an indication that the ministry of health’s messages were received as trustworthy… I think it’s a show of trust at the ministry of health”.*

Trust in the state and its institutions, or lack thereof, during the campaign was also framed in terms of the broader conversation on privatization processes, especially that of the national healthcare system, and their role in dismantling the sense of social solidarity. In an opinion piece published in the daily newspaper “Haaretz”, Ravit Hecht, one of the newspaper’s columnists, wrote:

*“A live vaccine is based on the principle of social responsibility and solidarity…. These values go against the existing socio-economic system…Once the state prefers to pull its hands from the life of the individual—in employment, welfare, education, retirement etc.—it lost control of his life, and following that his trust and willingness to enlist for social projects”* [15]

According to Hecht’s analysis, that was published at the height of the campaign and addressed the concerns over low vaccination adherence by the upper socio-economic classes, low adherence and lack of parents’ trust in the state and the ministry of health should be understood in a broader political context in which solidarity and social responsibility are no longer valued assets. The same state is now working to enlist these sentiments as part of the polio campaign. Summing up her argument on solidarity and social responsibility Hecht concludes*: “They (Israelis) learned to count only on themselves. So why is it (the government) messing with their heads now with social responsibility?”*

Issues of trust in the State and its institutions were of an even more complex nature when it came to the Bedouin communities that are marginalized by the state and its institutions, and are thus more suspicious of government policies. The initial outbreak was reported within Bedouin communities in the Negev. As a result, polio control efforts were first rolled out within these communities, and only later extended to the rest of the country in the form of a full vaccination campaign. Bedouin communities’ limited access to health services [13], especially in unrecognized Bedouin villages, and their tense relationship with the Israeli state, made trust an even more important issue during the polio campaign in Bedouin communities. Even outside the context of a government-ran polio vaccination campaign, health services offered by the state raise suspicion among the Bedouin population. A Bedouin nurse working in the south of Israel recounted some of the clashes she experienced in her routine work:

*“I often go with the outreach vehicle, in the heat, in the remote areas (gablaot), during Ramadan, and I experience violence. Our drivers experience violence. People say “we don’t want you here, what are you doing here? What do you want? Don’t come here, how many times have we told you not come here.” And then I try to convince them. Sometimes they are convinced, sometimes they aren’t convinced. It depends on who you are talking to, if they are willing to let you in or not.”*

The distrust among the Bedouins that is often associated with government sponsored health services extended to the polio campaign. Recounting the events of the polio campaign the same nurse talked about of her interactions with parents:

*“Parents came to ask me if this is the disease they are talking about on TV. They asked if the Jews are also being vaccinated, or just the Bedouins… because some people were spreading rumors that they (the government) want to kill Bedouins. They said “really, vaccinating the Jews?” and I said “I vaccinated [Jewish] children, what’s wrong with you? The same vaccine you have here, the same one there, no reason for fear”…. I have their trust, I’ve been there for 13 years”.*

In this case, the trust that was necessary for the campaign was not in the state and its institutions, that are not considered trustworthy, but in a nurse who has shown her commitment to the community for more than a decade. In many ways, the campaign proved that even trust is being individualized and in some cases no longer resides in the state, but in specific trustworthy individuals working for it. In these cases, the personal, trustworthy professional, has replaced the trust in the State.

This shift of trust from the State to individual health professionals was also evident in the Israeli Ministry of Health’s strategy of convening all relevant health institutions (e.g. Sick Funds, hospitals) and professional societies (e.g. Pediatrics, Family Physicians, Public Health) in order to convey a unified message. The Ministry sought to gain the trust of the professions so that all health professionals will convey the same message to the public, and not just those working for the ministry of health. Gaining the trust of the health professions first, was also one of the lessons learnt from a previous pandemic influenza campaign. The medical community’s ability to speak in one voice, and thus regain the public’s trust as individual professionals and as a profession (and not merely as the representatives of the State), was seen as an important strength of the campaign. This unity of message was able to harness the media to the campaign, as another pediatrician explained:

*“The media was unable to find any leading medical figure to come out against the campaign…. And once they (the media) saw that this is the situation, I think it was easier for them to back the campaign. And as a rule, the media was supportive.”*

However, the trust in this case, both the public’s and the media’s, was not primarily in the State and its institutions, but rather in individual physicians and nurses, and in the intimate relationships they had with the public.

### The paradox of success—balancing individual risks and collective benefits

In this state of affairs, where suspicion towards state institutions is on the rise and self-interest became much stronger, success in eradicating diseases becomes a challenge for health policy makers. In addition, public cooperation in vaccination and eradication campaigns is hindered by the paradox of success, namely that successful eradication campaigns change the profile of diseases, and thus make them less threatening to the population, and the campaigns to combat them less crucial. A public health physician, who has been working in both the public health sector and academia, explained in an interview:

*“We all knew that if there will be a case of polio it would be much easier to vaccinate afterwards. That’s the paradox of motives. If you are able to prevent [a disease] for years, you are able to prevent it now, identify an event very early on before there is actually a case, it is very hard to convince people to get vaccinated…. If you are successful [in eradication] you are shooting yourself in the leg”.*

Beyond the paradox of successful eradication, and the difficulties it created, public cooperation was also phrased in terms of the risks of an outbreak versus the risk associated with the vaccine. And while health professionals and the public phrased those risks in different terms, the need to address the balance of risk was prominent among both parents and health professionals. A public health physician, who was involved in the decision to initiate the campaign, addressed this complex problematic perception of risk:

*“I think the perception of real risk for me, and maybe even the director of the ministry of health and the minister, did not reach broader circles. I don’t know about physicians and nurses maybe yes, but not the public and that is a very dangerous thing… if we look at the media, there is the perception of relatively high risk associated with the vaccine, which is not justified, and way beyond the real risk…. It means that in a broader sense, people have an exaggerated perception of the risks associated with the vaccine, versus a perception of limited risk associated with the disease.”*

Launching a national vaccination campaign under these circumstances of conflicting perceptions of risk and benefit, and with no confirmed cases of polio in the population, required a unique set-up. And yet, while varying perceptions of risk were a major factor, interviewees addressed the sense of urgency that accompanied the vaccination campaign, and many of them described it in terms of a military operation. A mayor of a southern Israeli town that was at the center of the campaign described the atmosphere in his town:

*“I used everything [to get the word out], media, text messages, mosques, local media and journalists. The ministry of health published ads and distributed flyers to people… we met with physicians in town and we had like a war, like the military sets up for a new war, a war room and all those things. We had one enemy called polio, so we have to overcome it”.*

The war metaphor repeated in many of the interviews, and for some was also part of the explanation for the campaign’s success. A public health physician explained:

*“We know how to react well. And so if there’s an “operation” (mivtza), then the public knows how to react…. The public is used to it, the state is used to it, to know how to function during an operation in general, and vaccination campaigns are part of it…. The Israelis are very good at being recruited”.*

The war and military recruiting metaphors are also interesting as they bring to the fore an apparent contradiction between the growing individualism and the central role of the military in Israeli society, proving that there are still domains where a more communal perspective reigns in Israeli society (on the changing roles of the military in Israeli society see the work of Yagil Levy [19]).

While the atmosphere of emergency was prominent in the ministry’s action, and in some community leader’s responses, the call to vaccinate all children under the age of 8 years was not accepted by all. Moreover, the public reaction to the campaign was based on multiple layers of objection, refusal, and active suspicion directed at the state and its policies. Metaphors of military operations, state mechanisms, and duties not only evoke the familiar toolkit of Israeli citizenship as shaped by militarism, but also the rigid dichotomy between the state’s apparatus of coercions, and the citizens who feels threaten by uncertainty and thus foster practices of individual self-interest. In the context of this study, we wish to suggest how far these sentiments were from what was in fact needed from the public.

Furthermore, the military metaphor of public mobilization seems anachronistic in the face of the privatization process prevalent in Israeli society, as the threatening whip of the State loses its power in the era of massive liberalization. As a result, policy makers had to find another strategy to convince the public to cooperate with the goals of the campaign. For the first time in its history of vaccination campaigns, the Ministry of Health consulted a private public relations firm. The result was the campaign: “Two drops and the family is protected”, described below.

### The family in the campaign

Faced with the challenge of promoting an environmental vaccination campaign when there were no confirmed cases of polio in the population, and following the policy decision to initiate a campaign that was environmental in its nature, and not directed at the protection of children who were already vaccinated with IPV, the ministry of health had to craft a complex message. Solidarity was not understood as a strong motivator. Accordingly, and drawing from the family-oriented structure of Israeli society, a decision was made to craft a message targeting families. Kaliner et al [18] explained the decision:

*Many parents felt that OPV was a ‘social’ vaccine that builds on herd immunity and compensates for the small percentage of the population that has not received IPV, and felt that administering OPV to their IPV-vaccinated children is merely altruistic. Mindful of that, the message to the public was that the vaccine will protect their family members and close friends and not just the individual or the ‘environment’ or ‘society’ (*[18]*:3)*.

The narrow focus on one’s family, instead of a broader environmental message, or an altruistic message based on the welfare of a broad group of others, was apparent in the strategy employed by the ministry of health. The tension between a broad environmental message and a narrow focus on one’s family, and the benefit of honing the message towards a family oriented one, were clarified to the medical and public health community, and other professional groups, as the campaign was rolling out. A senior pediatrician who was involved in the design and execution of the campaign explained:

*“Environmental vaccine was the wrong term to use here, the right term to use, and ultimately the more emotionally appropriate one, was protecting the* ***family*** *, and not the environment. The environment is on the moon, it’s all the people resisting vaccinations, it’s all sorts of people I’m not interested in. In reality, everyone asks himself or herself “whom am I interested in?”. What I’m interested in is my family, the father, the grandfather, the grandmother, the mother—all the adults in the family. Maybe it wasn’t clear in the beginning [of the campaign].”*

The shift from a broad environmental message was not accidental, but a thought through decision reached with the help of a public relations firm contracted by the Ministry of Health. Commenting on the choice to use the family as the center of the campaign, one of the leading public health physicians in the county had said:

*“We were brainstorming with the PR firm which was behind the copywriting for the campaign, and came up with “2 drops and the family is protected”. This was the message we tried to convey, not the broad environmental message, not solidarity. Maybe 20-30 years ago when social solidarity in the cultural context was greater, this would have been the right thing to say—come get vaccinated like you join the Army. Get vaccinated and protect the homeland. But somehow we didn’t think such a message would work. So we tried to narrow the message, both when I did [media] interviews and the message itself, the focus was on the family.”*

The environmental message, turned family oriented, was very straightforward, as the most prominent tagline of the campaign stated: “Only two drops and the family is protected from the danger of polio”. Seen on every publication issued by the ministry of health, as well as on the ministry’s website, the message was clear—the polio campaign protects families—the family unit turned to be the relevant “circle of solidarity”. A senior ministry of health public health physician traced this vigorous family-oriented strategy when addressing campaign messaging in the media:

*In the media for example, the ones to deliver the messages were the pediatricians, and not the Ministry of Health staff who were seen as more detached and belonging to the state, and as such affiliated with the government’s guidelines and agendas. Pediatricians are seen as closer to field and maybe more reliable.*

According to this public health physician, the decision to use pediatricians instead of public health physicians was intentional due to their relationship with the families in "the field”. And so, while public health physicians might be the specialists when vaccination campaigns are concerned, pediatricians were the ones who have an established relationship with families, and thus were seen as representing families’ interests, instead of the interests of the state.

The Israeli focus on the safety of the family as a motive for vaccination becomes more evident when comparing the Israeli messaging with polio messages in other countries. While the theme of “two drops” is prevalent in polio eradication campaigns worldwide, the addition “and the family is protected from the danger of Polio” is an Israeli twist.

We see in this twist a crucial factor in understanding the tension between individualism and social solidarity. “The family” is an idiom which has its direct meaning in the sense of one’s particular family, but could also be substituted as a metaphor for Israeli society at large, where “soldiers are our children” and where “family” is a synonym for “community”. In fact, scholars have long indicated the central role of family in the sociological and political landscape of Israeli society [12, 16]. The permissive uses of reproductive technologies and genetic counseling in Israel indicate the central place of raising a family in the life courses of Israelis [1, 14]. The fact that marital laws in Israel are governed by religious laws indicates an effort to conserve the family as a fundamental unit in the Israeli sociological make-up. Furthermore, just as one cannot escape the family into which he or she is born, one cannot escape his or her religious identity as born to a Jewish mother. The conflation of religious identity, familial belonging and collective status in Israel is perhaps the central determinant of one’s identity. The family, therefore, stands—in Israel social polity—as the key factor in shaping Israeli collective life.

The ministry of health’s emphasis on the family aimed to the very heart of the Israeli sociological imagination. Its slogan introduced two key consensual items: “The Family” and “Protection”. Whereas “Protection” refers to the ever-present sense of threat in Israeli society, “The Family”, we argue here—stands for community. In other words, “The Family” remains a reference unit for society in an era of individualism. In a culture where society loses much of its political meaning, the family is introduced as a partial substitute for a society. Bonds of solidarity within Israeli society could only be tied by presenting it through the prism of the family in contemporary Israeli society. By doing so, the ministry of health sought public compliance, which cannot be reduced to the level of individualistic utilitarian motives.

Ultimately, the campaign achieved a 75% compliance rate. Although we have no actual data indicating that the remaining 25% refusal is due to individualism, we do see the use of the family metaphor as a key element in building a rather wide consensus regarding the importance of the campaign.

## Discussion and conclusion

Though vaccinations are usually considered a paradigm of bio-medical success, their use has frequently provoked fierce criticism and unparalleled opposition. Many current accounts of the dilemma stemming from the question of compliance to vaccination are based on a state-individual dichotomy. This interpretation draws from mainstream bioethical thinking, part of the liberal tradition that considers individual autonomy as central. This approach has aroused criticism and calls for bioethicists to take into account the social context and the unequal distribution of resources and power that frame peoples’ lives and health [8, 17, 27]. Even liberal philosophers like Norman Daniels have criticized the individualistic myopia of mainstream bioethical thinking, which does not examine “the broader institutional settings and policies that mediate population health” ([6]:23).

Following this criticism, our analysis calls to bring into account middle-range concepts such as the family as key factors in vaccination policy. Compliance or opposition to vaccination, as well as ways of persuasion and compulsion by the state, should be situated within a broader debate that is tied to questions of the limits of state power in the private sphere, such as family life, religious belief and health—often accentuated by ethnic tensions. Thus, the understanding of the ethical consideration embedded in the polio campaign must take into account not just individuals facing the state, or even individual families—it should also be considered within the broader social and political context, and mostly—as our analysis suggests—the social standing of the family i.e. as a metaphor bridging individualism and society. Such a broader approach in understanding vaccine compliance and opposition as more than an individualized decision is not limited to the Israeli case. Polio campaigns worldwide, as well as other vaccination efforts, are facing opposition that is far more complex than the mere individual decision to refuse vaccinations. These oppositions are tightly linked to both local historical contexts and global policy making processes and their implementation [4]. Identifying intermediate agencies, such as the family in our case, that bridge individual and collective identities, can help encourage the public to move beyond self-interest and advance public health objectives. Such agencies vary between different cultural contexts and can be the research objective of further investigation in the sociological context of vaccination policies and the efforts to persuade the public to move beyond self-interest to advance public health objectives.

The unique status of the family in the cultural history of vaccination policy in Israel can be inferred from the fact that historically, the vaccination of children in Israel, which comprise the overwhelming majority of vaccinations, are administered in Family Health Stations, or—as they are still called by most Israelis—*Tipat Chalav* (‘A Drop of Milk’) Clinics. This institution symbolized the special place assigned to the child, and the new mother raising the child, as a cornerstone in revitalizing and building the nation. Safeguarding the young child’s wellbeing was considered a central issue that demanded investment and forethought, taking strides to ensure adherence to the codes set down by public health personnel. These ideals, as shown in the 2013 polio campaign, have been changed: the family is not a clear metonym of the collective as before, yet it still bears the notion of a social unit, extending beyond the individual’s self-gain point of reference. In this sense, the family becomes the bridge between individualism and social solidarity.

## Footnotes

1 See the APHA code https://www.apha.org/~/media/files/pdf/membergroups/ethics_brochure.ashx and the EU statement on common values for health http://ec.europa.eu/health/strategy/principles/index_en.htm), including from a global health perspective (http://apps.who.int/iris/bitstream/10665/164576/1/9789240694033_eng.pdf

2 Bioethics is often identified with the four principles known as Georgetown Principlism (autonomy, justice, benevolence, and non-maleficence) which are associated with clinical bioethics or medical ethics that focus on the patient’s best interest. Social solidarity, however, pertains to a different register of bioethical conceptualization. Under this perspective, the focus of analysis is the interplay between the individual and the community.

3
http://www.ncbi.nlm.nih.gov/pubmed (Accessed 30 Aug 2015).

4 See Tasher et al. [25] for an analysis of the medical benefits of the campaign.
